# Post-Implantation Inflammatory Responses to Xenogeneic Tissue-Engineered Cartilage Implanted in Rabbit Trachea: The Role of Cultured Chondrocytes in the Modification of Inflammation

**DOI:** 10.3390/ijms242316783

**Published:** 2023-11-26

**Authors:** Ilya Klabukov, Dmitri Atiakshin, Evgenia Kogan, Michael Ignatyuk, Mikhail Krasheninnikov, Nickolay Zharkov, Anna Yakimova, Vyacheslav Grinevich, Pavel Pryanikov, Vladimir Parshin, Dmitry Sosin, Andrey A. Kostin, Peter Shegay, Andrey D. Kaprin, Denis Baranovskii

**Affiliations:** 1 National Medical Research Radiological Centre of the Ministry of Health of the Russian Federation, Koroleva St. 4, 249036 Obninsk, Russia; anna.prosovskaya@gmail.com (A.Y.);; 2 Department of Urology and Operative Nephrology, Patrice Lumumba Peoples Friendship University of Russia (RUDN University), 117198 Moscow, Russia; 3 Obninsk Institute for Nuclear Power Engineering, National Research Nuclear University MEPhI, 249031 Obninsk, Russia; 4 Scientific and Educational Resource Center for Innovative Technologies of Immunophenotyping, Digital Spatial Profiling and Ultrastructural Analysis, Patrice Lumumba Peoples Friendship University of Russia (RUDN University), 117198 Moscow, Russia; 5 Strukov Department of Pathological Anatomy, Sechenov First Moscow State Medical University (Sechenov University), 119991 Moscow, Russia; 6 Russian Child Clinical Hospital, Pirogov Russian National Research Medical University, 119571 Moscow, Russia; 7 National Medical Research Center of Phthisiopulmonology and Infectious Diseases of the Ministry of Health of the Russian Federation, 127473 Moscow, Russia; 8 Centre for Strategic Planning and Management of Biomedical Health Risks of the Federal Medical Biological Agency, 119121 Moscow, Russia; 9 Department of Biomedicine, University of Basel, Petersplatz 1, 4001 Basel, Switzerland

**Keywords:** chondrocytes, critical defect, decellularization, extracellular matrix, regenerative medicine, tissue engineering, trachea, xenogeneic transplantation

## Abstract

Immune responses to tissue-engineered grafts made of xenogeneic materials remain poorly studied. The scope of current investigations is limited by the lack of information on orthotopically implanted grafts. A deeper understanding of these processes is of great importance since innovative surgical approaches include the implantation of xenogeneic decellularized scaffolds seeded by cells. The purpose of our work is to study the immunological features of tracheal repair during the implantation of tissue-engineered constructs based on human xenogeneic scaffolds modified via laser radiation in rabbits. The samples were stained with hematoxylin and Safranin O, and they were immunostained with antibodies against tryptase, collagen II, vimentin, and CD34. Immunological and inflammatory responses were studied by counting immune cells and evaluating blood vessels and collagen. Leukocyte-based inflammation prevailed during the implantation of decellularized unseeded scaffolds; meanwhile, plasma cells were significantly more abundant in tissue-engineered constructs. Mast cells were insignificantly more abundant in tissue-engineered construct samples. Conclusions: The seeding of decellularized xenogeneic cartilage with chondrocytes resulted in a change in immunological reactions upon implantation, and it was associated with plasma cell infiltration. Tissue-engineered grafts widely differed in design, including the type of used cells. The question of immunological response depending on the tissue-engineered graft composition requires further investigation.

## 1. Introduction

The reconstruction of full-thickness defects in hollow epithelialized organs rich in endogenous microbiota is a complex problem that involves the issues of tissue revitalization, prompted epithelial colonization, vascular ingrowth, and the migration of immune cells into the inflammation zone [1,2]. The use of decellularized tissues with low residual DNA for tissue-engineered grafts represents the most common and widely studied approach to the repair of critical-size tracheal defects [3,4,5]. The application of xenogeneic tissues in clinical practice also contributes to solving donor deficiency in transplantology. Moreover, there is a need to study responses to the implantation of decellularized human tissues used as scaffolds since it has profound practical and fundamental importance for the planning of future studies [6,7]. 

In some cases, the normal physiological response to the implanted material led to the development of post-surgical complications [8,9]. The immune response that occurs during xenogeneic transplantation is a major problem limiting further progress in this field [10]. In this regard, studying the role of various factors that can potentially influence the increase or decrease in immune responses is of great importance. One of the questions to be addressed is whether seeding with cells can influence immunological responses and final outcomes.

Acute and chronic immune responses to the implantation of non-decellularized xenogeneic tissues in various animal models have been widely described. The implantation of non-devitalized porcine and bovine cartilage transplants in cynomolgus monkeys leads to chronic rejection responses, which are already evident within 1 month after transplantation [11]. The extensive chronic inflammatory process is characterized primarily by T-lymphocytes and macrophage infiltration. The implantation of decellularized lung scaffolds derived from α-Gal knockout pigs in a non-human primate model resulted in a greater proportion of infiltrating CD45+ cells, including CD3+ and CD8+ cytotoxic T cells [12]. The current understanding of cartilage homeostasis takes into account humoral and cellular immunity, which affects chondrocyte phenotype, regenerative capacity, and behavior. The role of these cells was predominantly studied relative to osteoarthritic conditions, and articular cartilage was the most demanded model, leaving gaps for further research with other cartilage types. ‘Assaulters’ of cartilage inflammation could be recognized in macrophages: macrostructures that are not phagocytosed elicit responses, attract other immune cells, and promote the development of cellular and humoral immunity [13]. Pro-inflammatory macrophage polarization leads to increased cartilage degeneration in patients with rheumatic disease and osteoarthritis [14,15]. 

Mast cells and macrophages play important roles in cartilage injury and repair [13,16], with mast cells promoting cartilage matrix degradation by inducing the production of MMPs and activating M1 macrophages to secrete inflammatory factors. The regulation of the cell-specific interactions of mast cells with materials could sufficiently improve implantation outcomes [17,18].

CD34+ cells are associated with the intensity of local neovascularization processes [19,20], during which intense angiogenesis prevents cartilage from maturing. Indeed, the inhibition of angiogenesis can promote stable cartilage formation [21,22]. Therefore, cartilage regeneration requires a different approach than that of vascular-dependent tissues.

Another key role is played by T cells: CD3+ T-cell aggregates and CD45+ cells have been found in the synovial membrane of patients with osteoarthritis and express activation markers [23]. In contrast to the well-studied inflammatory responses in articular cartilage, the immunology of tissue-engineered grafts is still in the shadows and requires further investigation due to the multiple and complex effects of the cells seeded on scaffolds, the physicochemical parameters of the scaffolds, and types of their modification. At the same time, the characteristics of immune responses to implantation of tissue-engineered constructs require further study due to the multifaceted and complex effects of cells seeded on scaffolds, physicochemical parameters of scaffolds, and types of their modification [24].

The study of post-implantation immune effects in tissue-engineered grafts could not be separated from the distinguishing of foreign body responses contributing to encapsulation and fibrosis [25]. The control of fibroblast proliferation and collagen accumulation has been attributed to vimentin, as evidenced by studies using a vimentin-deficient mouse model [26,27]. Vimentin has also been implicated in promoting fibrosis by stimulating the differentiation of mesenchymal cells into myofibroblasts [28]. For a better understanding of the immune responses, vimentin should be specifically detected in the analyzed grafts.

Previously, it was shown that the intravenous injection of adipose-derived mesenchymal stem/stromal cells (ADMSCs) can modulate the immune response after implantation of decellularized porcine bronchi in tracheal defects in rats. In particular, decreased numbers of CD8+ cytotoxic lymphocytes and CD163+ M2 regulatory macrophages were shown in the group receiving decellularized bronchi in combination with ADMSCs compared to decellularized bronchi alone [29]. Laser modification of scaffolds may also influence the immune response due to the artificial lacuna formation. Nürnberger et al. (2021) showed that murine cells were able to repopulate the empty lacunae in the crossed line engraved cartilage scaffold derived from the human and bovine knee [30].

The rabbit model remains relevant to humans in terms of the morphological features of the tracheal tissues [31], which makes this model applicable for investigation of the response to the implantation of tissue-engineered grafts (TEGs) used to restore tracheal defects. Relatively, only a few works are devoted to the problems of the orthotopic implantation of tissues from humans to rabbits. Thus, the rabbit’s bladder was reconstructed with the use of the human decellularized bladder [32], human acellular dermis (Alloderm) was used to reconstruct abdominal wall defects in rabbits [33], and human amniotic epithelial cells were transplanted into rabbit knee joints [34]. Rabbits have also been implanted with adenomatous cells of the human parathyroid gland [35], decellularized human skeletal muscle [36], decellularized human blood vessels [37], and decellularized human trachea with allogeneic rabbit cells [38]. These works are essential for the development of protocols for the evaluation of early and late implantation results.

**Figure 1:**
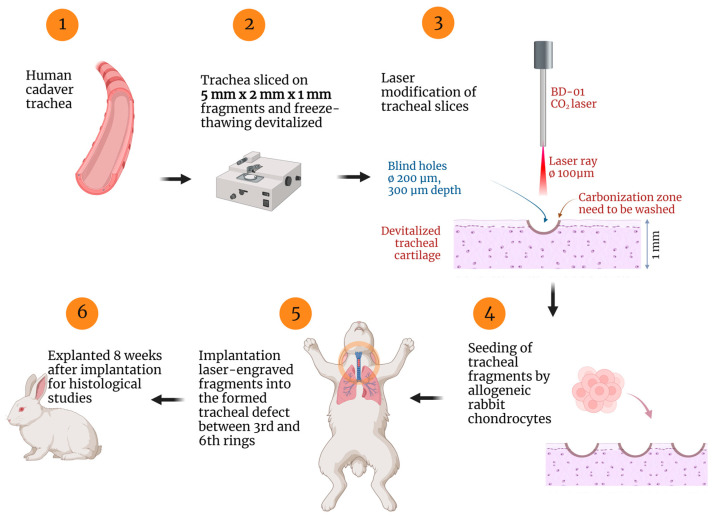
Design of the experimental study of implantation of tissue-engineered trachea based on laser-engraved cartilage scaffold seeded with allogeneic chondrocytes. Created with Biorender.com.

Previously, we showed that the implantation of a xenograft scaffold seeded with nasal chondrocytes in rabbits resulted in the functional repair of tracheal wall defects [39]. However, a deeper understanding of the reasons for the demonstrated outcomes requires further investigation of the tissue and cellular response to implanted grafts. The design of the performed study and the basic principle of tracheal tissue engineering based on laser-engraved cartilage enriched by allogeneic chondrocytes are shown in Figure 1.

In the present work, we describe the tissue and cellular responses following implantation of decellularized laser-engraved human tracheal cartilage scaffolds (DCS) and a tissue-engineered graft (TEG), based on the same DCS and seeded with rabbit nasal chondrocytes, into critical-size tracheal defects in rabbits.

## 2. Results

### 2.1. Histological Study

**Figure 2:**
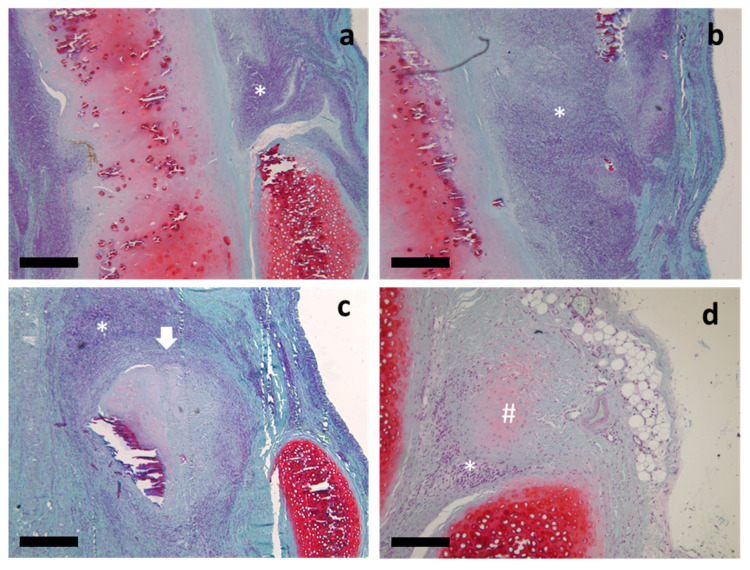
Histological study of DCSs and TEGs samples, both explanted eight weeks after orthotopic implantation, with Safranin-O and hematoxylin staining, under light microscopy: (**a**) general view of the DCS in the orthotopic position, longitudinal (sagittal) slices of the implant, showed infiltration of leukocytes (asterisk marked); (**b**) mucous membrane covering the DCS, and the submucosal infiltration of leukocytes (asterisk marked); (**c**) the TEG’s implantation zone with the presence of the fibrous capsule (arrow marked) with the presence of leukocytes (asterisk marked); (**d**) Safranin-O stained light pink, the focus of new cartilage tissue formation (hash marked) and infiltration of leukocytes (asterisk marked) in the TEG’s implantation zone. Scale bar 100 μm.

Our previous study showed complete mucosal regeneration at the implantation site in both the TEG and DCS groups [39]. In the present study, we performed an in-depth histological examination, which revealed extensive leukocyte infiltration in the submucosal layer in the DCS group (Figure 2a,b), while TEG was surrounded by a fibrous capsule with a reduced presence of leukocytes (Figure 2c,d).

In the TEG and DCS samples stained by hematoxylin and Safranin-O, it was found that the implant tissue was partially replaced by the recipient’s tissue, without areas of necrosis and fibrosis.

### 2.2. Plasma Cells Study

The presence of plasma cells was significantly higher in TEG specimens compared to the DCSs (294 vs. 50 cells per 1 mm^2^). The relative content of plasma cells was also higher in the TEG group (0.49% vs. 0.29%), However, this finding was not statistically significant.

### 2.3. Mast Cell Immunohistochemical Study

In an intact tissue, mast cells were detected as a limited population distributed predominantly in the tracheal mucosa and were less frequently detected in the connective tissue between the cartilaginous half-rings/islets.

**Figure 3:**
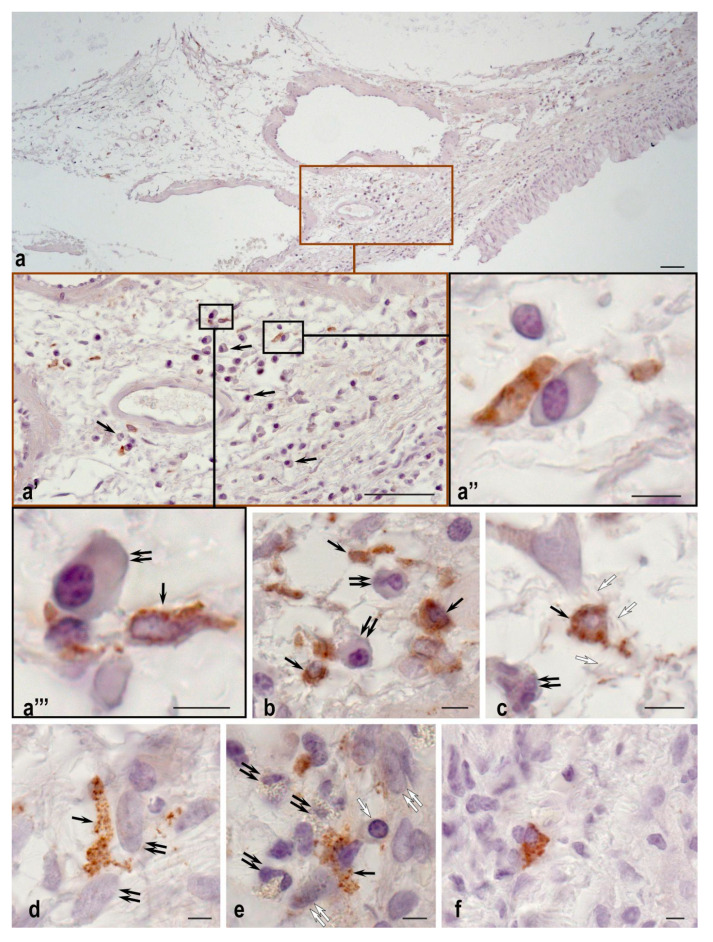
Tryptase-positive mast cells and plasma cells in a specific tissue microenvironment of the trachea, (**a**–**d**) TEG sample, (**e**,**f**) DCS sample. (**a**) An increase in the number of mast cells (stained brown) and plasma cells (indicated by a black arrow) in the membranes of the trachea. (**a’**,**a’’**) enlarged fragments; (**a’’’**) Attachment of a mast cell (indicated by a black arrow) with signs of tryptase secretion to a plasma cell (indicated by a double black arrow). (**b**) Colocalization of mast cells (indicated by a black arrow) with plasma cells (indicated by a double black arrow); (**c**) Mast cell (indicated by a black arrow), neutrophil granulocyte (indicated by double black arrow), and fibrous component of extracellular matrix (indicated by a white arrow) in the tracheal stroma; (**d**) Interaction of a mast cell (indicated by a black arrow) and fibroblast (indicated by a double black arrow); (**e**) Interaction of a mast cell (indicated by a black arrow) with granular leukocytes (indicated by a double black arrow), a plasma cell (indicated by a white arrow), and fibroblasts (indicated by a double white arrow); (**f**) Mast cell in the stroma of the trachea. Scale bar: (**a**,**a’-a’’’**) 50 µm, (**b**–**f**) 5 µm.

The implantation of both TEG and DCS into the tracheal defect resulted in an increase in the number of plasma cells and mast cells in the peri-implantation area (Figure 3).

The number of mast cells in the peri-implantation tissue increased approximately equally in both TEGs and DCSs samples, but was insignificantly higher in the TEG group (13 vs. 5 cells per 1 mm^2^). Mast cells’ secretory activity was also increased.

Interestingly, more extensive colocalization of mast cells and plasma cells was observed in the trachea implanted with TEG, whereas mast cells were more often colocalized with fibroblasts when a DCS was implanted.

### 2.4. Vimentin Immunohistochemical Study

**Figure 4:**
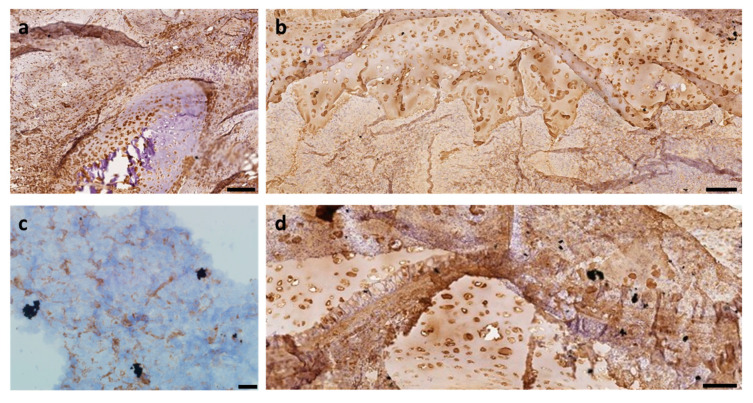
Vimentin staining: (**a**) TEG sample, (**b**) DCS sample; Collagen II staining: (**c**) TEG sample, (**d**) DCS sample. Scale bar: (**a**) 50 µm, (**b**) 100 µm, (**c**) 10 µm, (**d**) 50 µm.

IHC staining revealed a fibrous capsule with thin vimentin-positive fibers around implants in both TEG and DCS groups (Figure 4a,b). Vimentin staining was weaker in DCS samples compared to TEG samples.

### 2.5. Collagen Type II Immunohistochemical Study

Collagen II staining of the TEG samples showed collagen-positive newly formed cartilage tissue in the area of TEG implantation; the matrix of TEG was positively stained (Figure 4c). In the DCS samples, collagen was absent or weakly stained, with only a mild synthesis of collagen II in the peripheral part of the scaffold (Figure 4d).

### 2.6. CD34+ Immunohistochemical Study

**Figure 5:**
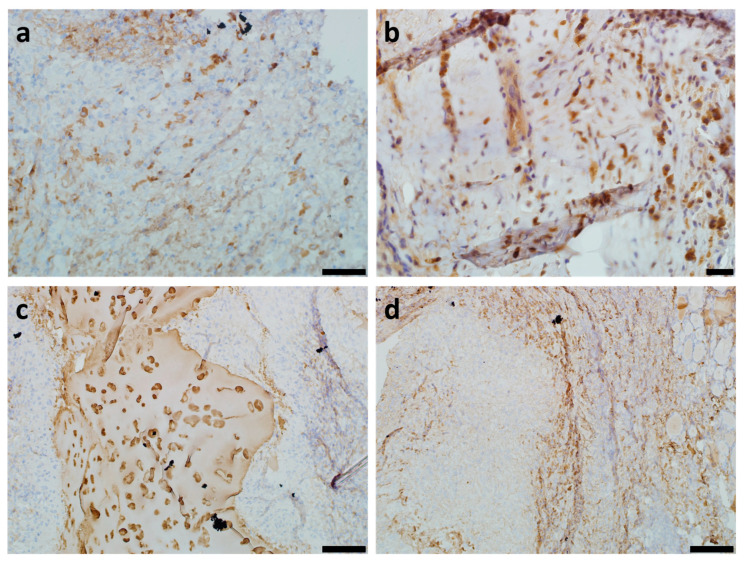
Staining of the explanted samples for CD34+: (**a**,**b**) TEGs, (**c**,**d**) DCSs. Scale bar: (**a**) 50 µm, (**b**) 5 µm, (**c**,**d**) 100 µm.

IHC staining revealed a significant CD34+ response in the TEG samples, as well as newly formed vessels in the submucosal layer and angiogenesis in the granulation tissue (Figure 5a,b). In the DCS scaffold samples, angiogenesis was observed in the submucosal layer, but almost no angiogenesis was observed in the scaffold area (Figure 5c,d).

Differences in various parameters between groups are presented in Table 1.

## 3. Discussion

Experimental transplantation of human tissues into model animals is a relatively rare technique primarily used only in immunodeficient mice [39,40,41,42]. In fact, scientists are currently faced with the problem of adverse events and side effects after clinical application of cell therapies, implantation of tissue-engineered constructs [42,43], and drug-loaded polymeric scaffolds [44,45,46,47]. Therefore, the detailed mechanisms of immunological responses to tissue-engineered grafts remain mostly undiscovered.

Although decellularization is a well-studied technique for biomaterial processing [48], the tissue-specific reactions to the implantation of decellularized materials have not been investigated [49]. Das et al. (2021) showed that decellularized xenogeneic goat cartilage preserved the bioactivity and integrity of the matrices, which also favored in vitro stem cell proliferation, and chondrogenic differentiation after implantation into rabbits [50].

Xenogeneic tissues’ transplantation promotes angiogenesis and tissue regeneration by activated TREM2+ macrophages [51]. Cell administration was found to be important for the outcome of restoration. Previously, it was shown that seeding the biocompatible scaffold with nasal chondrocytes promotes regenerative changes in cartilage tissue [52]. Willers et al. (2005) suggest that an autologous chondrocyte-seeded collagen membrane is an effective method for the treatment of focal osteochondral knee injury in rabbits, where the cells had a positive effect on cartilage repair [53]. The study showed that the cell concentration had no effect on histological outcomes, suggesting the existence of an effective low-dose chondrocyte cell therapy.

**Figure 6:**
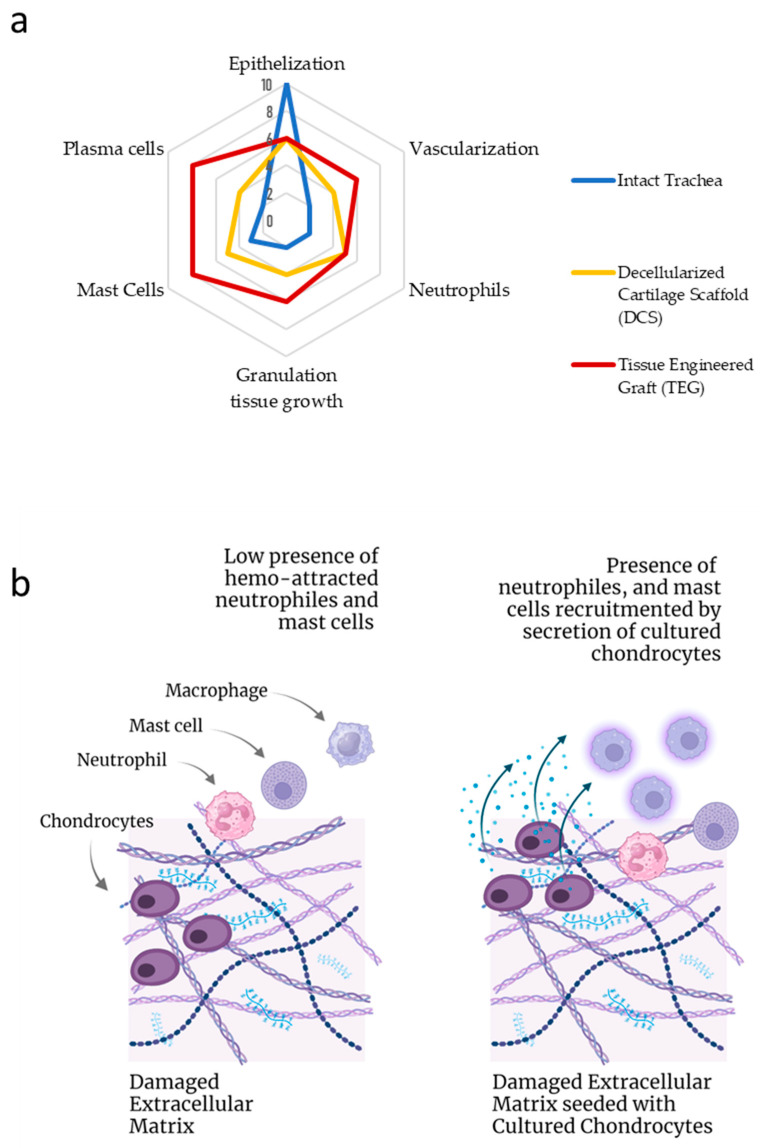
(**a**) Radar plot of the semi-quantitative evaluation of the results of implantation of tissue-engineered and devitalized grafts of tracheas in comparison with intact tissue; (**b**) Visualization of the hypothetical mechanism of participation of cultured chondrocytes seeded on the devitalized scaffold in the recruitment of plasma cells and mast cells through secretion activity caused by seeding on damaged surfaces. Created with Biorender.com.

In our study, the TEG and DCS groups were characterized by different immunological responses. TEG implantation was associated with a higher number of plasma cells in comparison to DCS samples. Cartilage-infiltrating plasma cells are known to be high producers of IL-6, which regulates the microenvironment of chondrocytes and stimulates them to produce MMPs [54]. Increased expression of MMPs directly leads to intensive cartilage bioresorption. At the same time, a more pronounced leukocyte infiltration was observed in the decellularized scaffold samples, which may indicate the potential presence of lymphocytes as a response to the decellularized material. In order to summarize and visualize the obtained outcomes of our study, a radar plot was created to present the comparative fields on a single scale, as shown in Figure 6a.

Surprisingly, allogeneic cells seeded on xenogeneic scaffolds did not suppress graft inflammation, but altered the balance between immune cell populations. Human implantation of decellularized caprine conchal cartilage demonstrated the biocompatible, robust, and non-toxic properties of the matrix. The viability and safety of the material, both in an animal model and human pre-application in the actual site, were shown [55]. The reason for this may be the role of seeded cells as adjuvants for inflammatory responses caused by damaged cartilage scaffolds (Figure 6b).

**Figure 7:**
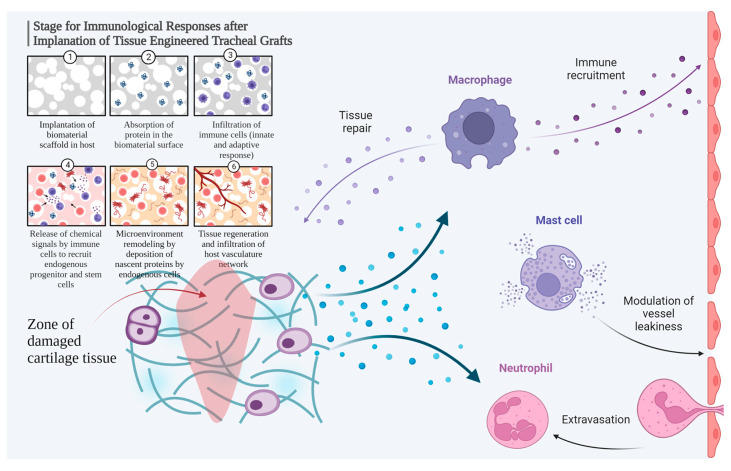
Immunity following the cartilage graft implantation. Created with BioRender.com.

Up-to-date, cell-free regenerative approaches are promising techniques for transplantation with low immunological impact [56,57]. Decellularized xenogeneic scaffolds combined with autologous chondrocytes induced neocartilage and better structural restoration at 8 weeks after transplantation into rabbit knee articular cartilage defects [58], so the authors concluded that a decellularized xenogeneic cartilage matrix with laser-engraved micropores provides an ideal scaffold for the functional reconstruction of articular cartilage. However, the short-term observation period does not allow for the detection of the chronic immunological responses. Currently, the outcomes of tracheal reconstruction with decellularized scaffolds remain unclear [59], especially considering that many of the biocompatible materials may be toxic or poorly tolerated and induce inflammatory phenomena or rejection [60]. In the study by Bomhard et al. (2019), considering groups with or without cell seeding, no complete cartilage healing occurred ad integrum, while cartilage formation from the perichondrium was more irregular than from the seeded scaffold [61]. Common mechanisms and stages of immunological responses after implantation of tissue-engineered tracheal grafts are shown in Figure 7.

To determine immune responses, we focused on the assessment of mast cells immunostained for tryptase. Among pre-formed mediators, tryptase is the most abundant protein in the secretory granules of human mast cells, accounting for up to 25% of the total protein content of the cell [62,63]. Tryptase has a wide range of biological activities and has been shown to regulate immunogenesis, serve as a component of innate immunity, facilitate toxin inactivation, and regulate the state of internal stromal elements through extracellular matrix remodeling, including the stimulation of fibrous structure formation and progression of fibrosis in preclinical animal models [64]. The effects of tryptase on the tissue microenvironment can be differentiated into pro- or anti-inflammatory [63,65,66,67]. In general, tryptase initiates inflammation by increasing the permeability of capillary walls and enhancing the migration of neutrophils, eosinophils, basophils, and monocytes beyond the microcirculatory bed [68]. Tryptase is intimately involved in angiogenesis processes [69,70], participating in several mechanisms of growth and differentiation of new blood vessels, including inflammation [66,71,72,73]. The effects of tryptase on fibroblast cells manifest as the activation of their migration, mitotic division, and stimulation of collagen synthesis, setting the stage for fibrotic outcomes [74,75]. Tryptase has a high affinity for PAR-2 receptors, potentiating the development of inflammation [76]. The localization of these receptors on various cells of specific tissue microenvironments can induce leukocyte migration, edema, and other responses. In addition, the activating effect of tryptase on PAR-2 receptors of afferent neurons can lead to neurogenic inflammation and the formation of pain syndrome. An important regulatory mechanism of tryptase in potentiating inflammation is the sustained upregulation of PAR-2 receptor expression in various tracheal cells. Tryptase can activate the secretion of pro-inflammatory mediators by cells of specific tissue microenvironments into the extracellular matrix, resulting in increased background levels of certain cytokines and chemokines [76,77].

Regarding the interaction between mast cells and plasma cells, it should be noted that mast cells can have activating effects on plasma cells. In addition, mast cells have been shown to enhance the proliferation of B lymphocytes and their differentiation into mature plasma cells, as well as to stimulate the synthesis of immunoglobulins by plasma cells [78]. The immunological response to tissue-engineered cartilage derived from auricular chondrocytes and a PLLA scaffold suggested that chondrocytes in tissue-engineered cartilage constructs could regulate the actions of host-derived macrophages by expressing factors associated with immune privilege [79].

In our study, the presence of somatic cells seeded on a DCS scaffold resulted in a leukocyte-based inflammatory response in contrast to the plasma cell-based responses observed in TEG samples.The main limitation of the study was the small number of animals used. This factor does not allow for statistically significant results and the conclusions can only be presented semi-quantitatively. Because immunostaining for macrophage subpopulations was not performed, the conclusion that M2 macrophages are involved in the response to tissue-engineered grafts remains unclear. Therefore, the study provides only preliminary results in the emerging field of the detailed investigation of inflammatory responses.

In addition, the use of a single endpoint of observation did not allow us to see the dynamics of immune responses. Long-term follow-up to assess whether observed immunologic changes persist or evolve over time would provide valuable insights into the dynamics of immune responses in tissue engineering.

## 4. Materials and Methods

### 4.1. Samples Preparation

Histological and immunohistochemical studies were carried out on explanted samples of decellularized laser-perforated human tracheal cartilage (DCS samples) and on explanted TEG samples (n = 4 for each group). Both types of explants were previously implanted in a critical-sized tracheal defect in rabbits [39]. The process of creating samples and conducting a surgical experiment was described in detail previously [33,39]. In brief, human tracheal cartilage was decellularized using a freeze–thaw method followed by laser perforation with the use of a ‘Trotec Speedy 300’ (Trotec Ltd., Marchtrenk, Austria) laser engraver. Samples of laser-engraved cartilage were divided into 2 groups: decellularized laser-perforated tracheal cartilage (control or DCS group, scaffold), and decellularized laser-engraved tracheal cartilage seeded with cultured allogeneic rabbit nasal chondrocytes (TEG group). The model of a critical tracheal defect was created by cutting off four rings of the rabbit tracheal wall (the 3rd, 4th, 5th, and 6th rings). TEGs and DCSs scaffolds were implanted into the tracheal defects and fixated with Prolene 7-0 sutures. Both groups were explanted 8 weeks after implantation [39].

### 4.2. Histological and Immunohistochemical Studies

Paraffin tissue sections (5 μm-thick for histological and 2 μm-thick for immunohistochemical staining) were deparaffinized with xylene and rehydrated according to the standard protocol [80]. Sections were prepared on a HistoCore AUTOCUT rotary microtome (Leica Biosystems, Wetzlar, Germany).

For histologic analysis, slices were stained with hematoxylin and Safranin-O according to the standard protocol [39]. For immunohistochemical analysis, the deparaffinized sections were processed for antigen retrieval at 95 °C (30 min) in a specialized R-UNIVERSAL buffer (Aptum Biologics Ltd., Southampton, SO16 8AD, UK). Blocking of endogenous Fc receptors before incubation with primary antibodies was not performed, as recommended [81]. After blocking endogenous peroxidase activity, primary antibodies against tryptase (#ab2378, dilution 1:2000), collagen type II (Thermo Fisher Scientific, Waltham, MA, USA; dilution 1:500) and vimentin (Thermo Fisher Scientific, Waltham, MA, USA; dilution 1:1000), were applied and incubated overnight at +4 °C. Visualization of primary antibodies in tissue structures was performed using horseradish peroxidase-conjugated secondary antibodies (Amplistain™ anti-mouse 1-step HRP, SDT GmbH, Kraichtal, Germany), and the DAB Peroxidase Substrate Kit (#SK-4100, Vector Laboratories, Burlingame, CA, USA) detection system. Nuclei were counterstained with Mayer’s hematoxylin (HK-G0-DL01, Biowitrum, Stockholm, Sweden), and sections were mounted in a permanent mounting medium. Images were processed using ImageJ software version 1.54e (ImageJ, NIH) with the Fiji plugin.

### 4.3. Microscopy

Stained sections were examined using a motorized microscope ZEISS Axio Imager.Z2 with Zeiss alpha Plan-Apochrom 100×/1.46 Oil DIC M27 objectives, Zeiss Plan-Apochrom 150×/1.35 Glyc DIC Corr M27 objectives, and the ZEISS Axiocam 712 color camera (Carl Zeiss Vision, Jena, Germany). The acquired images were processed using the software packages Zen 3.0 Light Microscopy Software Package, ZEN Module Bundle Intellesis & Analysis for Light Microscopy, and ZEN Module Z Stack Hardware (Carl Zeiss Vision, Germany).

Planimetric analysis to determine the number of mast cells per unit area of tracheal tissue, as well as the absolute number of mast cells and other tracheal cells, was performed using the QuPath software version 0.4.4 (https://qupath.github.io/, assessed on 9 May 2023) after scanning the microslides with a Leica Scanscope Aperio Cs2 microscope (Leica, Wetzlar, Germany) [82].

### 4.4. Statistics

The Mann–Whitney U test is used to compare differences between two independent groups. Statistical analysis was performed using GraphPad Prism version 8.4.3 for Windows (GraphPad Software, Boston, MA, USA). A radar plot was performed using a united semi-quantitative 10-point scale for morphometry. All derived numeric values were re-calculated, and qualitative values were transferred in the scale. Differences were considered significant at a *p*-value < 0.05.

### 4.5. Ethics

The human tracheal cartilage obtained from a cadaver donor was used as a scaffold. Human cadavers that met the following criteria: ≥18 years old at the moment of death, and no trachea-bronchial diseases, infections, disorders, or malignancies. All procedures were conducted with the approval of the Local Ethics Committee of Sechenov University (15 July 2015, Protocol No. 07-15).

## 5. Conclusions

The most striking finding of our study is the different inflammatory responses observed in the TEG and DCS groups. Leukocyte-based inflammation predominated during the implantation of decellularized unseeded scaffolds, whereas plasma cells were significantly more abundant in TEGs. The described results may provide evidence for a sophisticated physiological bridge between the cell seeding of scaffolds used in tissue engineering and the development of a specific type of immune response.

## Figures and Tables

**Table 1:** Differences between tissue-engineered constructs and decellularized tracheal cartilage scaffolds.

Parametre	Tissue-Engineered Constructs (TEG), Experimental Group	Decellularized Unseeded Tracheal Cartilage (DCS), Control Group
Fibrosis	Focal fibrosis	N/R
Leukocyte infiltration	Lower presence of leukocytes	Extensive leukocyte infiltration in the submucosal layer
Mast cell count	13 cells per 1 mm^2^	5 cells per 1 mm^2^
Mast cell co-localization	More frequent co-localization of mast cells and plasma cells	More frequent co-localization of mast cells and fibroblasts
Plasma cell count	294 cells per 1 mm^2^	50 cells per 1 mm^2^
Relative content of plasma cells	0.49%	0.29%
Vimentin	0.19% (SD 0.05%) More pronounced vimentin staining	0.13% (SD 0.08%) Weaker vimentin staining.
Collagen type II	0.54% (SD 0.25%) Collagen-positive TEG matrix and neoformed cartilage tissue in the area of TEG implantation	0.34% (SD 0.19%) Collagen staining is poorly expressed or absent; mild synthesis of collagen II in the peripheral part of the scaffold only
CD34+ cells	0.18% (SD 0.01%) Distinct positive response; newly formed vessels in the submucosal layer and angiogenesis in the granulation tissue	0.10% (SD 0.03%) Angiogenesis was observed in the submucosal layer only; no angiogenesis was observed or weakly expressed in the scaffold area

## Data Availability

Full-size scans of all specimens stained with Safranin-O and hematoxylin and immunostained with tryptase, vimentin, and CD34+ are available on request.
